# Timely Initiation of Argatroban Improves Prognosis in Patients With Acute Ischemic Stroke Attributed to Large Artery Atherosclerosis: A Cohort Study

**DOI:** 10.1002/brb3.70799

**Published:** 2025-08-27

**Authors:** Qiu Liu, Xiao Wu, Dashuai Huang, Yanqing Wang, Haibo Wu, Xinchen Zhu

**Affiliations:** ^1^ Department of Neurology Nanshi Hospital of Nanyang Nanyang China; ^2^ Department of Neurology Xuanwu Hospital Beijing China; ^3^ Department of Neurology Nanyang Central Hospital Nanyang China

**Keywords:** argatroban, acute ischemic stroke, large artery atherosclerosis, timely initiation

## Abstract

**Introduction:**

Previous studies have demonstrated the benefits of anticoagulant therapy in acute ischemic stroke (AIS) of large artery atherosclerosis (LAA) etiology, although no prior research has examined the impact of the timing of anticoagulation initiation in this population.

**Methods:**

A retrospective cohort study was conducted to assess the effect of early argatroban administration on clinical outcomes in AIS with LAA. Patients were stratified into an early administration group and a late administration group based on the time from stroke onset to argatroban initiation: ≤24 h and >24 h. The primary outcome was the proportion of favorable outcome, defined as modified Rankin Scale (mRS) score 0–2 at 90 days. Secondary outcomes included the proportion of patients achieving mRS 0–1 at 90 days, the mRS score at 90 days, and changes in National Institutes of Health Stroke Scale (NIHSS) score from baseline to 7 days or discharge. Safety outcomes comprised symptomatic intracranial hemorrhage (sICH), early neurological deterioration (END) during hospitalization, and organ hemorrhage within 90 days. Adjustments for potential confounders were performed using logistic regression.

**Results:**

From February 2022 to February 2024, 401 AIS patients treated with argatroban were enrolled. After excluding patients with non‐LAA etiologies, thrombolysis, and follow‐up loss, 55 patients received argatroban ≤24 h after stroke onset, and 98 received it >24 h. At 90 days, 43.6% of the early administration group achieved favorable outcomes versus 27.6% of the later administration group (*p* = 0.024). One sICH occurred in the later administration group. END was observed in one (1.8%) patient in the early administration group and five (5.1%) in the later administration group.

**Conclusion:**

This is the first study to demonstrate that timely argatroban initiation may improve clinical outcomes in thrombolysis‐naive patients with LAA AIS. However, the results should be interpreted with caution due to the study's retrospective design and limited sample size.

## Graphical abstract

This is the first study to demonstrate that early argatroban initiation (≤24 h) may improve 90‐day favorable outcomes in thrombolysis‐naive patients with acute ischemic stroke of large artery atherosclerosis etiology, though the results warrant cautious interpretation due to its retrospective design and limited sample size.

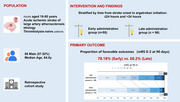

## 1 Introduction

Stroke is the second leading cause of global morbidity and mortality, and more than 80% of these are ischemic strokes (Saini et al. 2021). Large artery atherosclerosis (LAA) contributes to ∼25% of acute ischemic stroke (AIS). Despite advancements in reperfusion therapies, including thrombolysis and mechanical thrombectomy (Badhiwala et al. 2015; Majoie et al. 2023), many patients with LAA‐related AIS remain ineligible for these interventions due to contraindications or delayed presentation (Ye et al. 2022). Argatroban, a direct thrombin inhibitor, has been used for anticoagulation in AIS patients (Hou et al. 2021; Zhang et al. 2024) and demonstrates potential to enhance neurological function and outcomes in patients experiencing early neurological deterioration (END) (Hou et al. 2021).

Preliminary studies suggest that early combination therapy with argatroban and antiplatelet agents (aspirin or clopidogrel) may benefit LAA‐related AIS patients by mitigating progressive neurological deficits (Li et al. 2022; Wang et al. 2021; Xu et al. 2022; Yan et al. 2025). However, the optimal timing for argatroban administration following stroke onset remains undefined. While early anticoagulant initiation might decrease early recurrence risk and improve functional recovery, concerns persist regarding potential increased intracranial hemorrhage risk.

In this study, we aimed to determine whether administration of argatroban within 24 h of stroke onset improves functional outcomes and maintains comparable safety profiles compared to later administration in this patient population.

## 2 Methods

### 2.1 Study Design and Population

This was a retrospective, observational cohort study at the Cerebrovascular Center, Nanshi Hospital of Nanyang. The study protocol was approved by the ethics committee of Nanshi Hospital of Nanyang (KY‐2024‐IEC‐030). Given the retrospective nature of this observational cohort study, all data were anonymized. Consequently, the Ethics Committee waived the requirement for informed consent. All procedures were carried out in strict accordance with relevant guidelines and regulations.​

The inclusion criteria were as follows: (i) age ranging from 18 to 80 years, (ii) diagnosis of acute ischemic stroke confirmed by computed tomography (CT) or magnetic resonance imaging (MRI), and (iii) treatment with argatroban. Patients were excluded if they met any of the following criteria: (i) had a stroke type other than LAA according to the Stop Stroke Study Trial of Org 10172 in Acute Stroke Treatment (SSS‐TOAST) classification criteria (Ay et al. 2005), such as cardioaortic embolism, small‐artery occlusion, other determined causes, or undetermined causes; (ii) had a modified Rankin Scale (mRS) score ≥3 before the onset of the current stroke; (iii) received thrombolysis or endovascular treatment; (iv) were lost to follow‐up; (v) had cancer or any other severe comorbid disease; or (vi) were pregnant.

### 2.2 Interventions

Argatroban was continuously infused at a dosage of ∼60 mg/day during the first 2 days. The infusion rates of argatroban were adjusted to a target activated partial thromboplastin time (APTT) of 1.75 times the baseline value (±10%) (Sugg et al. 2006). Subsequently, it was administered twice a day at a dosage of 20 mg/day for 5 days. Meanwhile, either aspirin (100 mg/day) combined with clopidogrel (75 mg/day) or aspirin alone (100 mg/day) was also administered.

### 2.3 Data Collection and Outcomes Evaluation

The data utilized in this study were extracted from the electronic dataset collected between February 2022 and February 2024. Information was retrieved from electronic medical records, encompassing demographic characteristics, clinical variables, and outcome measures.

The primary outcome was defined as the proportion of patients with a favorable outcome, which was defined as an mRS score of 0–2 at 90 days post‐stroke. The mRS scores were obtained through face‐to‐face interviews or telephone calls. The mRS ranges from 0 (indicating no symptoms) to 6 (representing death). Secondary outcomes included the proportion of patients with an mRS score of 0–1 at 90 days, the mRS scores at 90 days, the change in National Institutes of Health Stroke Scale (NIHSS) score from baseline to 7 days or at discharge, and the NIHSS scores at discharge. Safety outcomes were symptomatic intracranial hemorrhage (sICH), END during hospitalization, and organ hemorrhage at 90 days. sICH was defined as any extravascular blood within the brain or cranium that was associated with clinical deterioration, as evidenced by an increase of four or more points in the NIHSS score, or that led to death and was identified as the primary cause of neurological deterioration (Hacke et al. 2008). END was defined as an increase of ≥1 point in motor function or an increase of ≥2 points in the total NIHSS score (Kwon et al. 2014).

### 2.4 Statistical Analysis

The mean ± SD or median (interquartile range) was used to describe continuous variables depending on the normality of the distribution. Categorical variables were described with *n* (%). For the comparison of continuous variables, the independent samples *T*‐test or Mann–Whitney *U* test was used. For the comparison of categorical variables, chi‐square test was used. Univariate and multivariate logistic regression was used to adjust for potential confounders and estimate the odds ratio (OR) and 95% confidence interval (CI) for the primary outcome. All tests of significance were two‐tailed, and *p* < 0.05 was considered statistically significant. The data analysis was performed using IBM SPSS Statistics 25.0.

## 3 Results

### 3.1 Baseline Characteristics

**FIGURE 1:**
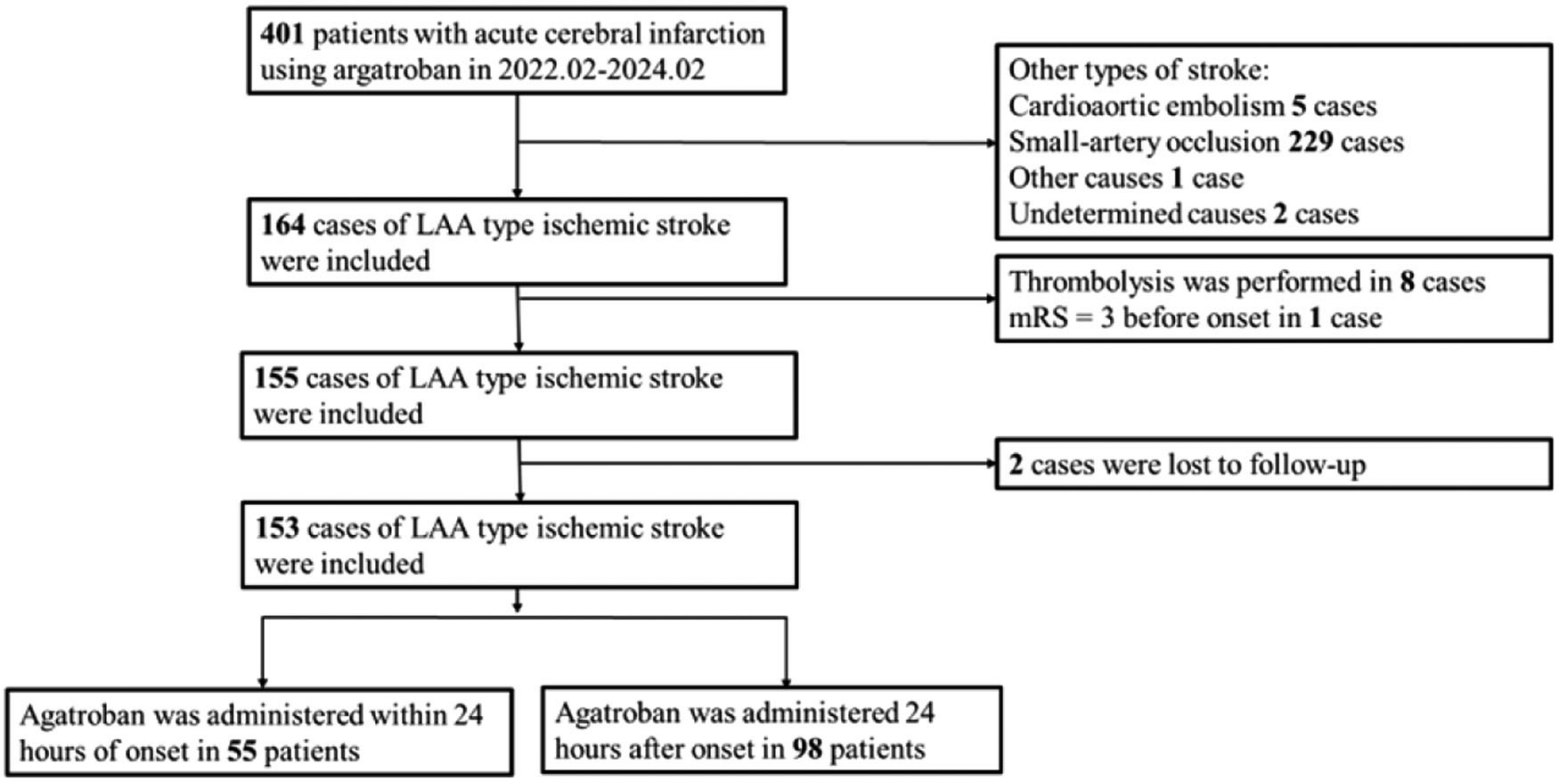
Study flow chart of the patient selection process. LAA: large artery atherosclerosis.

From February 2022 to February 2024, a total of 401 ischemic stroke patients treated with argatroban were initially enrolled. As depicted in Figure 1, after excluding 248 patients (237 with non‐LAA stroke types, eight underwent thrombolysis, one with a pre‐stroke mRS score of 3, and two lost to follow‐up), the final study cohort consisted of 153 patients with LAA type ischemic stroke. Among these, 55 patients received argatroban within 24 h of stroke onset (early administration group), and 98 patients received the drug more than 24 h after stroke onset (late administration group). The median age of the entire cohort was 64 years (interquartile range [IQR]: 54–72), with 57.5% being male. Hypertension (69.9%) and diabetes (26.8%) were prevalent comorbidities. The median baseline NIHSS score was 3 (IQR: 2–5). As shown in Table 1, there were no significant differences in baseline characteristics between the two groups (all *p* > 0.05).

**TABLE 1:** Characteristics of the participants.

Variables	Total (*n* = 153)	Early administration group (*n* = 55)	Late administration group (*n* = 98)	Statistic	*p*
Age, M (Q₁, Q₃)	64.00 (54.00, 72.00)	67.00 (57.00, 73.50)	62.00 (54.00, 70.00)	*Z* = −1.53	0.126
Gender, *n*(%)				*χ* ^2^ = 0.02	0.901
Male	88 (57.52)	32 (58.18)	56 (57.14)		
Female	65 (42.48)	23 (41.82)	42 (42.86)		
BMI, M (Q₁, Q₃)	25.95 (23.38, 27.73)	25.95 (22.52, 27.51)	25.87 (23.46, 27.77)	*Z* = −0.62	0.535
SBP, M (Q₁, Q₃)	150.00 (140.00, 160.00)	150.00 (140.00, 160.00)	150.00 (140.00, 160.00)	*Z* = −0.10	0.917
Smoking, *n*(%)	44 (28.76)	19 (34.55)	25 (25.51)	*χ* ^2^ = 1.40	0.236
Drinking, *n*(%)	28 (18.30)	12 (21.82)	16 (16.33)	*χ* ^2^ = 0.71	0.399
Hypertension, *n*(%)	107 (69.93)	41 (74.55)	66 (67.35)	*χ* ^2^ = 0.87	0.351
Diabetes, *n*(%)	41 (26.80)	13 (23.64)	28 (28.57)	*χ* ^2^ = 0.44	0.508
Coronary artery disease, *n*(%)	17 (11.11)	5 (9.09)	12 (12.24)	*χ* ^2^ = 0.35	0.551
Time from stroke onset to argatroban use, M (Q₁, Q₃)	27.50 (13.00, 54.50)	10.50 (7.75, 13.75)	49.50 (28.00, 96.50)	*Z* = −10.25	<0.001*
Baseline NIHSS, M (Q₁, Q₃)	3.00 (2.00, 5.00)	3.00 (2.00, 4.50)	3.00 (2.00, 6.00)	*Z* = −1.20	0.231
Site of infarction, *n*(%)				*χ* ^2^ = 1.43	0.232
Anterior circulation	126 (82.35)	48 (87.27)	78 (79.59)		
Posterior circulation	27 (17.65)	7 (12.73)	20 (20.41)		
mRS score before stroke, *n*(%)				*χ* ^2^ = 1.05	0.591
0	123 (80.39)	46 (83.64)	77 (78.57)		
1	17 (11.11)	6 (10.91)	11 (11.22)		
2	13 (8.50)	3 (5.45)	10 (10.20)		
Antiplatelet Therapy, *n*(%)				*χ* ^2^ = 3.10	0.079
Dual antiplatelet therapy	130 (84.97)	43 (78.18)	87 (88.78)		
Single antiplatelet therapy	23 (15.03)	12 (21.82)	11 (11.22)		

Abbreviations: ‐: Fisher exact; mRS, modified Rankin Scale; NIHSS, National Institutes of Health Stroke Scale; Q₁: 1rd Quartile, Q_3_: third Quartile; *Z*: Mann–Whitney test.

*p < 0.05.

### 3.2 Outcomes Assessment

**FIGURE 2:**
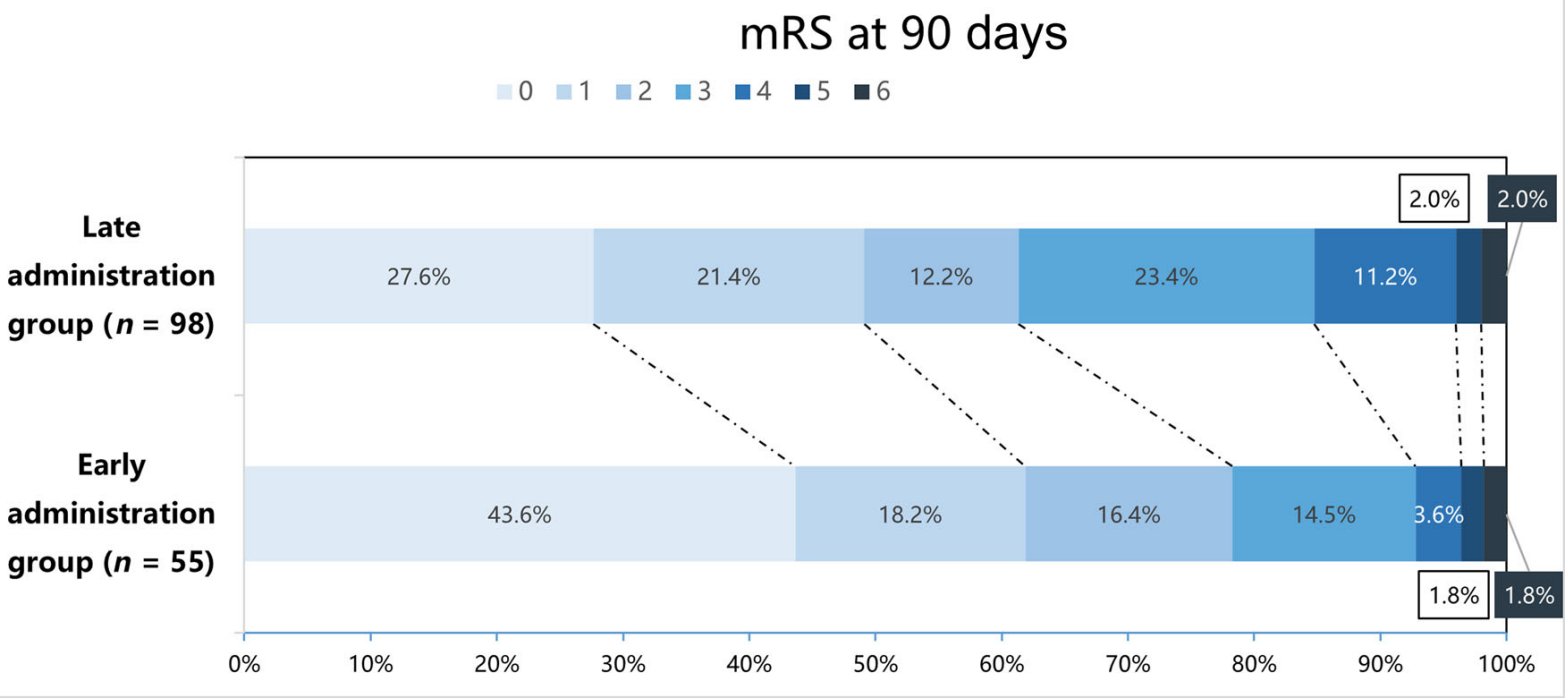
Distribution of 90‐day mRS scores. mRS, modified Rankin Scale.

As presented in Table 2, at 90‐day follow‐up, 78.18% (43/55) of patients in the early argatroban administration group achieved favorable outcomes (mRS of 0–2 at 90 days), compared to 60.20% (59/98) in the late administration group (*p* = 0.024). The median mRS score at 90 days was also lower in the early group (median = 1, IQR 0–2) than in the late group (median = 2, IQR 0–3; *p* = 0.029), and the distribution of 90‐day mRS scores between the two groups is shown in Figure 2. Although the proportion of patients with excellent functional outcomes (mRS 0–1) at 90 days was higher in the early group (61.82% [34/55] vs. 48.98% [48/98]), this difference did not reach statistical significance (*p* = 0.127). Changes in NIHSS scores from baseline to 7 days or discharge were similar between the two groups (median change: 1.0 vs. 2.0; *p* = 0.330), and there was no significant difference in NIHSS scores at discharge (median: 1.0 vs. 2.0; *p* = 0.221).

**TABLE 2:** The efficacy and safety outcomes between two groups.

Variables	Total (*n* = 153)	Early administration group (*n* = 55)	Late administration group (*n* = 98)	Statistic	*p*
Primary outcome					
mRS 0–2 at 90 days, *n*(%)	102 (66.67)	43 (78.18)	59 (60.20)	*χ* ^2^ = 5.12	0.024*
Secondary outcomes					
mRS 0–1 at 90 days, *n*(%)	82 (53.59)	34 (61.82)	48 (48.98)	*χ* ^2^ = 2.33	0.127
mRS at 90 days, M (Q₁, Q₃)	1.00 (0.00, 3.00)	1.00 (0.00, 2.00)	2.00 (0.00, 3.00)	*Z* = −2.18	0.029*
NIHSS changes at 7 days or discharge, *M* (Q₁, Q₃)	2.00 (1.00, 2.00)	1.00 (0.50, 2.00)	2.00 (1.00, 2.00)	*Z* = −0.97	0.330
NIHSS score at discharge, *M* (Q₁, Q₃)	2.00 (0.00, 4.00)	1.00 (0.00, 3.00)	2.00 (0.00, 4.00)	*Z* = −1.22	0.221
Safety outcomes					
Symptomatic intracranial hemorrhage, *n*(%)	1 (0.65)	0 (0.00)	1 (1.02)	—	1.000
END during hospitalization, *n*(%)	6 (3.92)	1 (1.82)	5 (5.10)	*χ* ^2^ = 0.33	0.569
Organ hemorrhage at 90 days, *n*(%)	2 (1.31)	1 (1.82)	1 (1.02)	—	1.000

Abbreviations: END: early neurological deterioration; ‐: Fisher exact; mRS, modified Rankin Scale; NIHSS, National Institutes of Health Stroke Scale; Q₁: 1rd Quartile, Q_3_: third Quartile; *Z*: Mann–Whitney test.

*p < 0.05.

For safety outcomes, sICH occurred in only one patient (1.02%) in the late administration group, with no cases reported in the early group (*p* = 1.000). The incidence of END during hospitalization was less frequent in the early group (1.82% [1/55] vs. 5.10% [5/98]; *p* = 0.569). Organ hemorrhage rates at 90 days were low and comparable between groups (1.82% [1/55] vs. 1.02% [1/98]; *p* = 1.000).

### 3.3 Logistic Regression Analysis

**FIGURE 3:**
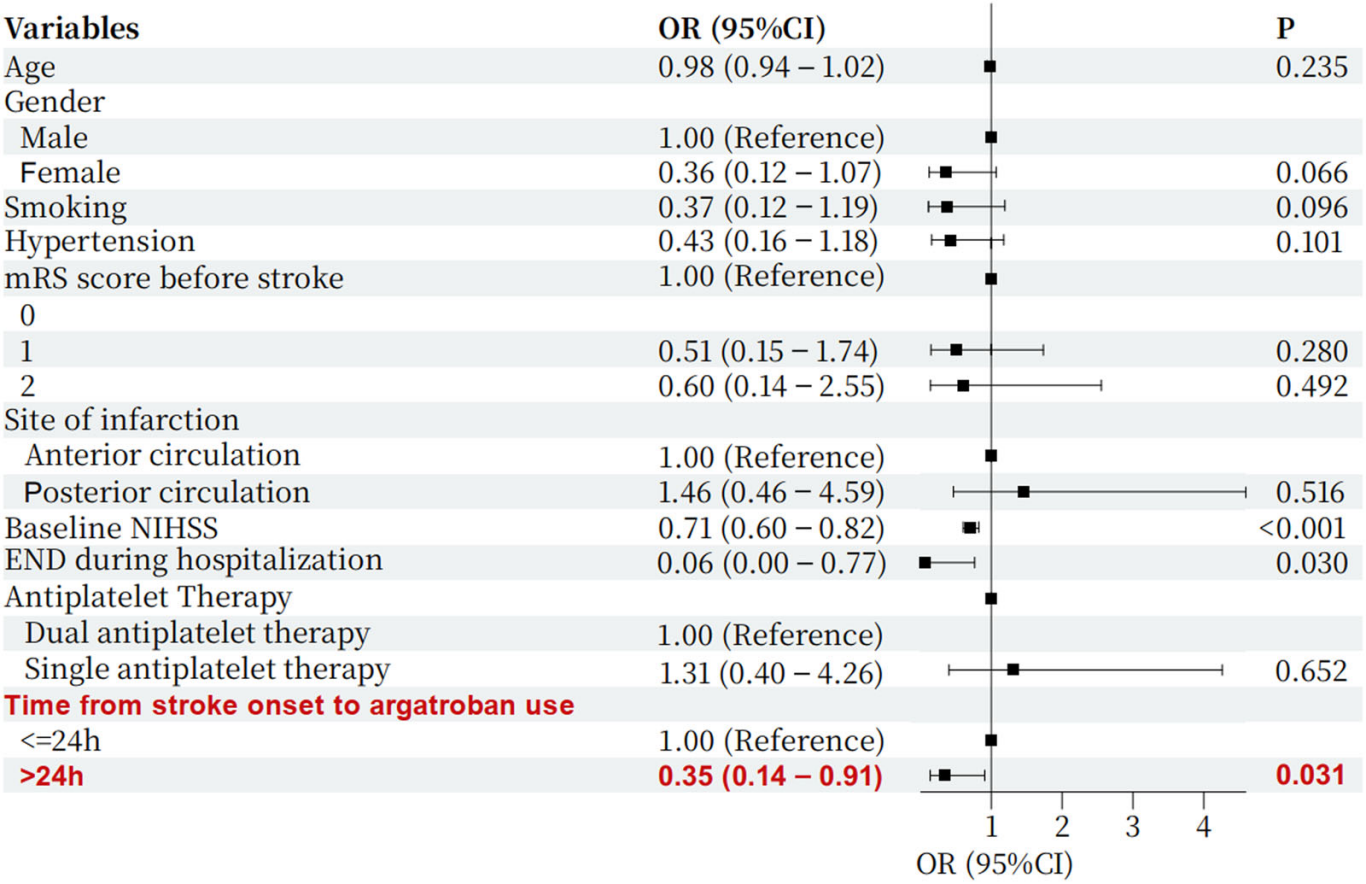
Multivariate logistic regression forest plot of favorable outcome.

Logistic regression analysis was conducted to adjust for potential confounding factors. The early argatroban administration was associated with a decreased likelihood of poor outcomes, with an adjusted OR of 0.42 (95% CI: 0.20–0.90, *p* = 0.026). Other variables significantly associated with poor outcomes included hypertension (*p* = 0.049), pre‐stroke mRS score (*p* = 0.025), baseline NIHSS score (*p* = 0.001), and END during hospitalization (*p* = 0.031). Multivariate logistic regression analysis further confirmed that early argatroban administration (OR = 0.35, 95% CI: 0.14–0.91, *p* = 0.031) was an independent predictor of favorable outcomes (Table 3, Figure 3).

**TABLE 3:** Univariate and multivariate logistic regression between the two groups (argatroban administration ≤24 h vs. >24 h).

Variables	Unadjusted					Adjusted				
	*β*	SE	*Z*	*p*	OR (95%CI)	*β*	SE	*Z*	*p*	OR (95%CI)
Age	−0.02	0.01	−1.67	0.095	0.98 (0.95–1.00)	−0.02	0.02	−1.19	0.235	0.98 (0.94–1.02)
Gender										
Male					1.00 (reference)					1.00 (reference)
Female	−0.40	0.35	−1.15	0.249	0.67 (0.34–1.32)	−1.01	0.55	−1.84	0.066	0.36 (0.12–1.07)
Smoking	−0.33	0.37	−0.88	0.378	0.72 (0.35–1.49)	−0.98	0.59	−1.66	0.096	0.37 (0.12–1.19)
Hypertension	−0.80	0.41	−1.97	0.049*	0.45 (0.20–0.99)	−0.84	0.51	−1.64	0.101	0.43 (0.16–1.18)
mRS score before stroke										
0					1.00 (Reference)					1.00 (reference)
1	−0.53	0.53	−0.99	0.322	0.59 (0.21–1.67)	−0.68	0.63	−1.08	0.280	0.51 (0.15–1.74)
2	−1.35	0.60	−2.24	0.025*	0.26 (0.08–0.84)	−0.51	0.74	−0.69	0.492	0.60 (0.14–2.55)
Site of infarction										
Anterior circulation					1.00 (reference)					1.00 (reference)
Posterior circulation	0.21	0.46	0.45	0.653	1.23 (0.50–3.04)	0.38	0.58	0.65	0.516	1.46 (0.46–4.59)
Baseline NIHSS	−0.32	0.07	−4.59	<0.001*	0.73 (0.63–0.83)	−0.35	0.08	−4.42	<0.001*	0.71 (0.60–0.82)
END during hospitalization	−2.40	1.11	−2.16	0.031*	0.09 (0.01–0.80)	−2.80	1.29	−2.17	0.030*	0.06 (0.00–0.77)
Antiplatelet therapy										
Dual antiplatelet therapy					1.00 (reference)					1.00 (reference)
Single antiplatelet therapy	0.16	0.49	0.32	0.749	1.17 (0.45–3.05)	0.27	0.60	0.45	0.652	1.31 (0.40–4.26)
Time from stroke onset to argatroban use										
≤24h					1.00 (reference)					1.00 (reference)
>24h	−0.86	0.39	−2.23	0.026*	0.42 (0.20–0.90)	−1.04	0.48	−2.16	0.031*	0.35 (0.14–0.91)

Abbreviations: CI: confidence interval; OR: odds ratio.

*p < 0.05.

## 4 Discussion

To the best of our knowledge, this is the first study to elucidate the potential benefits of administering argatroban within 24 h (≤24 h) of the onset of LAA‐type AIS. Compared to delayed administration (more than 24 h), the early administration group demonstrated a significantly higher proportion of favorable outcome at 90 days, while maintaining comparable safety profiles. This finding fills the evidence gap regarding the impact of anticoagulation timing on LAA stroke outcomes (Li et al. 2022; Yan et al. 2025) and provides critical insights for clinical decision‐making. Although current guidelines remain controversial regarding argatroban use in non‐cardioembolic stroke (Al‐Salihi et al. 2024; Cheng et al. 2024), this study supports its value in early intervention for the LAA subgroup, particularly in patients who did not receive thrombolysis or thrombectomy. Furthermore, the study reinforces the “time is brain” principle, suggesting that early intervention may optimize clinical outcomes.

The improved prognosis associated with early argatroban administration may stem from multifaceted mechanisms. First, the pathogenesis of LAA stroke involves local thrombosis and distal embolism following atherosclerotic plaque rupture (Rosário and Fonseca 2023). As a direct thrombin inhibitor, argatroban rapidly suppresses thrombin activity, reducing fibrin deposition and platelet aggregation, thereby stabilizing culprit plaques and preventing thrombus extension (Sun et al. 2024; Xie et al. 2024). Second, early anticoagulation may improve microcirculatory perfusion, preserving the ischemic penumbra—a critical factor for LAA patients, where large artery stenosis exacerbates hemodynamic compromise (Shi et al. 2022). Notably, the lower incidence of END in the early administration group (1.8% vs. 5.1%) aligns with argatroban's potential to attenuate thrombin‐mediated inflammatory responses and endothelial injury (Maclean et al. 2022; Ueshima et al. 2000; Zhang et al. 2024). Additionally, argatroban's short half‐life (30–50 min) (Xie et al. 2024) enables rapid anticoagulation with controllable bleeding risks, making it suitable for the acute‐phase treatment of LAA stroke.

The application of argatroban in ischemic stroke treatment remains a subject of extensive debate. While some studies have highlighted its potential as adjunctive therapy (such as in combination with dual antiplatelet agents or thrombolytics) (Xu et al. 2022, 2022), trials like The Argatroban Plus Recombinant Tissue‐Type Plasminogen Activator for AIS study (ARAIS) failed to confirm additional benefits when combined with alteplase (Chen et al. 2023). Clinically, argatroban is more commonly used for END or high thrombotic burden, with no consensus on optimal timing. Our study further validates the feasibility of early argatroban therapy, showing a low hemorrhagic risk, which aligns with previous safety data (Zhang et al. 2024). However, most existing evidence derives from non‐randomized designs, and the small sample size may limit generalizability. Future research should conduct multicenter randomized controlled trials to validate the 24‐h treatment window and refine dosing protocols, and also integrate imaging biomarkers (such as perfusion imaging or thrombus burden scores; Lin et al. 2025) to identify patients most likely to benefit from argatroban treatment.

This study has several notable limitations. First, the retrospective design may introduce selection bias and confounding, potentially affecting the validity of the results. Second, the relatively small sample size and lack of subgroup analysis (such as differentiating between anterior and posterior circulation; Cui and Chen 2025) may restrict the generalizability of the findings. Third, we did not perform perfusion imaging, plaque imaging, or assessment of relevant laboratory markers (e.g., D‐dimer, fibrinogen). Finally, all enrolled patients were from a single region in China, and differences in ethnic backgrounds, lifestyle habits, and medical practice patterns may affect the extrapolation of our findings to other populations (e.g., Western cohorts). Future multicenter studies encompassing diverse populations, along with comprehensive imaging and laboratory assessments, will be necessary to validate and expand upon these findings.

## 5 Conclusion

Early administration of argatroban within 24 h of stroke onset is associated with better functional outcomes at 90 days. These findings suggest that timely initiation of anticoagulant therapy is crucial for improving patient outcomes after ischemic stroke. Further large‐scale, prospective studies are warranted to confirm these findings.

## Author Contributions

**Qiu Liu**: conceptualization, data curation, project administration, visualization, validation, writing–original draft. **Xiao Wu**: conceptualization, formal analysis, investigation, methodology, visualization, validation, writing–review and editing, writing–original draft. **Dashuai Huang**: data curation, investigation, methodology. **Yanqing Wang**: data curation. **Haibo Wu**: data curation, software. **Xinchen Zhu**: conceptualization, funding acquisition, project administration, supervision, resources.

## Conflicts of Interest

The authors declare no conflicts of interest.

## Peer Review

The peer review history for this article is available at https://publons.com/publon/10.1002/brb3.70799.

## Data Availability

The data that support the findings of the study are available from the corresponding author on reasonable request.
